# Mannose reduces fructose metabolism and reverses MASH in human liver slices and murine models *in vivo*

**DOI:** 10.1097/HC9.0000000000000671

**Published:** 2025-03-21

**Authors:** John G. Hong, Joshaya Trotman, Yvette Carbajal, Poulomi Dey, Mariel Glass, Victoria Sclar, Isaac L. Alter, Peng Zhang, Liheng Wang, Li Chen, Mathieu Petitjean, Dipankar Bhattacharya, Shuang Wang, Scott L. Friedman, Charles DeRossi, Jaime Chu

**Affiliations:** 1 Department of Pediatrics, Icahn School of Medicine at Mount Sinai, New York City, New York, USA; 2 Diabetes, Obesity, and Metabolism Institute, Icahn School of Medicine at Mount Sinai, New York City, New York, USA; 3 Institute of Cardiovascular Sciences, State Key Laboratory of Vascular Homeostasis and Remodeling, Department of Physiology and Pathophysiology, School of Basic Medical Sciences, Peking University, Beijing, China; 4 PharmaNest Inc., Princeton, New Jersey, USA; 5 Department of Medicine, Division of Liver Diseases, Icahn School of Medicine at Mount Sinai, New York, New York, USA

**Keywords:** fibrosis, fructose, mannose, MASH, stellate cell

## Abstract

**Background:**

Fibrosis drives liver-related mortality in metabolic dysfunction–associated steatohepatitis (MASH), yet we have limited medical therapies to target MASH-fibrosis progression. Here we report that mannose, a simple sugar, attenuates MASH steatosis and fibrosis in 2 robust murine models and human liver slices.

**Methods:**

The well-validated fat-and-tumor MASH murine model for liver steatosis and fibrosis was employed. Mannose was supplied in the drinking water at the start (“Prevention” group) or at week 6 of the 12-week MASH regimen (“Therapy” group). The *in vivo* antifibrotic effects of mannose supplementation were tested in a second model of carbon tetrachloride (CCl_4_)-induced liver fibrosis. A quantitative and automated digital pathology approach was used to comprehensively assess steatosis and fibrosis phenotypes. Mannose was also tested *in vitro* in human and primary mouse hepatocytes conditioned with free fatty acids alone or with fructose, and human precision-cut liver slices from patients with end-stage MASH cirrhosis.

**Results:**

Oral mannose supplementation improved liver fibrosis *in vivo* in both fat-and-tumor MASH and CCl_4_ mouse models, as well as in human precision-cut liver slice MASH samples. Mannose also reduced liver steatosis in fat-and-tumor MASH mice, and in human and mouse hepatocytes *in vitro*. Ketohexokinase, the main enzyme in fructolysis, was decreased with mannose in whole mouse liver, cultured hepatocytes, and human precision-cut liver slices. Removal of fructose or overexpression of ketohexokinase each abrogated the antisteatotic effects of mannose.

**Conclusions:**

This study identifies mannose as a novel therapeutic candidate for MASH that mitigates steatosis by dampening hepatocyte ketohexokinase expression and exerts independent antifibrotic effects in 2 mouse models and human liver tissue slices.

## INTRODUCTION

The era of drug therapy for metabolic dysfunction–associated steatotic liver disease (MASLD) is on the horizon. There are many candidates in the pipeline, with a select few in advanced stages of clinical trials and one with U.S. Food and Drug Administration approval.^1^ However, developing an effective therapy has been challenging due to the complex MASLD/metabolic dysfunction–associated steatohepatitis (MASH) pathophysiology, which includes: (1) metabolic dysregulation with fat accumulation and lipotoxicity, (2) inflammation, and (3) consequent fibrosis.^2^ Therapeutic approaches have largely been siloed with the spotlight currently focused on steatosis.^3^ However, fibrosis drives liver-related morbidity and mortality in MASH,^4^ and developing an effective therapy to attenuate fibrosis directly is paramount.^5^

**FIGURE 1:**
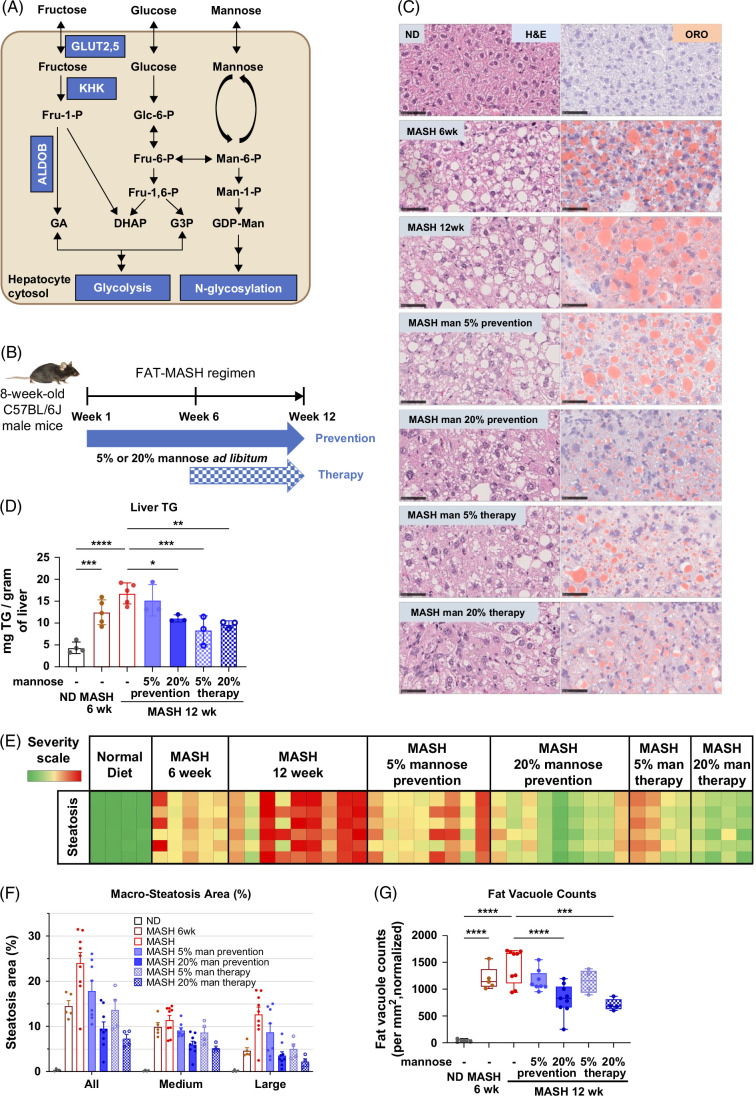
Mannose supplementation in FAT-MASH mice alleviates steatosis and lipid profile. (A) Graphical outline of fructose, glucose, and mannose metabolism in a hepatocyte. (B) Graphical outline of the FAT-MASH regimen and mannose treatments. (C) Representative images of H&E and ORO staining from whole liver sections. Scale bar: 50 µm. (D) Hepatic TG analyses. (E) Heat chart of macrosteatosis quantification (FibroNest) in mouse livers from all treatment groups (n=4–9). Each column represents an individual mouse. Each row represents a unique parameter of steatosis—rows 1, 2: area ratio and normalized count; rows 3, 5: medium vacuoles (6–18 um diameter), and rows 4, 6: large vacuoles (>18 µm diameter). (F) Bar chart depicting quantification of steatosis area (%) for all medium or large vesicles. Individual mice are indicated by points, and statistical significance is shown in Supplemental Table S1, http://links.lww.com/HC9/B935. (G) Box plot showing the normalized density (count per mm^2^) of fat vacuoles for each treatment group (n=4–9). Results are expressed as mean ± SD with analysis by one-way ANOVA with Šidák post hoc test or Student *t* test (***p<0.001 and ****p<0.0001). Abbreviations: FAT-MASH, fat-and-tumor MASH; H&E, hematoxylin and eosin; KHK, ketohexokinase; MASH, metabolic dysfunction–associated steatohepatitis; ND, normal diet; ORO, Oil Red O; TG, triglycerides.

An effective antifibrotic therapy should also mitigate the upstream metabolic effects of fibrosis.^4^^,^^5^ Fructose has been shown to contribute to the rise in obesity globally,^6^ and there is a direct and positive association between fructose intake and MASLD risk,^7^^–^^9^ hepatic fatty acid synthesis,^10^ and MASH-fibrosis stage.^11^ Clinical trials demonstrate that isocaloric fructose restriction improves hepatic fat content in adults^12^ and children.^7^ Therefore, targeting intracellular fructose metabolism is timely, essential, and promising. Ketohexokinase (KHK) catalyzes the first committed step in fructose metabolism and is a key and feasible target (Figure 1A). KHK has been linked to MASLD progression; it is increased in MASH mouse models^13^^,^^14^ and patients,^14^ and in preclinical studies, inhibition of KHK was effective in mitigating MASH steatosis.^15^^,^^16^ Recently, phase 2 studies using a KHK inhibitor in adults with MASLD have shown reduced hepatic steatosis with acceptable safety and tolerability (NCT03256526, NCT03969719).^17^^,^^18^

Mannose is a simple sugar and C_2_ epimer of glucose that is metabolically linked to fructose (Figure 1A). Its therapeutic potential has been long overlooked. Oral mannose supplementation is well-tolerated and commercially available at a low cost, making it an appealing therapeutic option. Recently, mannose supplementation has been recognized to play disease-modifying roles in obesity,^19^ diabetes,^20^ and cancer.^21^^,^^22^ We have previously shown that mannose supplementation can directly dampen the activation of HSCs, the major driver of liver fibrosis, *in vitro* and decrease fibrogenesis *in vivo*.^23^ Hu et al^24^ demonstrated an antisteatotic effect of mannose in a mouse model of alcoholic liver disease. Seeking to leverage the previous findings that suggest mannose may be both antifibrotic and antisteatotic in other liver diseases, we examined mannose as a potential novel therapy in MASH.

## METHODS

### Mice

Eight-week-old male C57BL/6J mice (Jackson Laboratory) were housed 3–5 per cage and kept with a 12-hour dark/light cycle. All procedures were performed according to the Animal Care and Use Committee of Icahn School of Medicine at Mount Sinai protocols (IACUC-2015-0050).

### Fat-and-tumor MASH model

Control mice received a normal chow diet (LabDiet, Rodent diet 20, #5053) with RO water ad libitum. MASH mice were placed on fat-and-tumor MASH (FAT-MASH) regimen of 21.1% fat, 41% sucrose, and 1.25% cholesterol in the chow (Teklad diets, 120528) with sugar water of 23.1 g/L d-fructose (Sigma-Aldrich, F0127) and 18.9 g/L d-glucose (Sigma-Aldrich, G8270) and 0.2 μL/g of body weight CCl_4_ (Sigma-Aldrich, 289116-100ML) i.p. injections 1×/week.^25^ Mannose 5% or 20% (w/v) was supplemented in drinking water (Thermo Fisher Scientific, AAA1084). Mannose consumption was calculated by multiplying mannose concentrations of 5 g/100 mL for 5% and 20 g/100 mL for 20% groups by average water intake measured in milliliters mouse/day.

### CCl_4_ model

Mice received i.p. injections of 0.05 mL 20% CCl_4_ or corn oil as vehicle control 3×/week for 4 weeks.^26^ Mice were treated with 5% or 20% mannose in drinking water at week 0 or 2.

### Histology

Samples were cut from whole livers, fixed overnight in 10% buffered formalin, and paraffin-embedded, sectioned, and stained with hematoxylin and eosin or Sirius Red (Abcam, ab150681) by the Mount Sinai Biorepository and Pathology CoRE. For Oil Red O (ORO) staining (Thermo Fisher Scientific, AAA1298914), the fresh liver was embedded in O.C.T. Compound (Thermo Fisher Scientific, 23-730-571), flash frozen, and sectioned (10 μm). Samples were blinded and scored for fibrosis severity based on METAVIR guidelines. ImageJ was used for Sirius Red quantification.

### Lipid measurements

Liver triglycerides (TG) were extracted as previously described,^27^ and measured using an Infinity TG kit (Thermo Fisher Scientific, TR22421).

### Serum analysis

Blood collection was done via cardiac puncture, and serum was prepared by centrifugation at 2000*g* for 10 minutes at 4 °C. Serum ALT, AST, and bilirubin levels were measured by VRL Diagnostics.

### Digital pathology and artificial intelligence

Sections were stained with Picrosirius Red and imaged with the Aperio Digital Pathology system. FibroNest was used to quantify phenotypes as previously described.^28^ Percent macrosteatotic area of non-fibrotic tissues is computed using medium (6–18 μm), large (>18 μm), or combined (all). Normalized vacuole counts (count/mm^2^) are used as markers for the generation and growth of vacuoles. Small vacuoles (<6 μm) were not used as algorithms performed poorly by other forms of “white space” around hepatocytes.

### Bulk RNA sequencing

Bulk liver RNA sequencing (RNA-seq) was performed by Novogene (N=3/group) and NovaSeq PE150 (Illumina). Quality was confirmed (Q30≥91%) and aligned to mouse reference GRCm39 using STAR RNA-seq aligner^29^ and counts matrix generated with featureCounts function from the Rsubread package. Counts were processed for differential expression analysis and visualization using the standard workflow of the DESeq2 package (version 1.42) in R (version 4.3) as outlined in the DESeq2 vignette. Principal component analysis plots were generated using regularized log-transformed counts. Heatmaps for fructose metabolic and uptake genes were generated using size-factor-normalized counts of genes included in GO_BP_FRUCTOSE_METABOLIC_ PROCESS (GO:0006000) and GO_BP_FRUCTOSE_TRANSMEMBRANE_TRANSPORT (GO:0015755) gene sets.

### Quantitative PCR analysis

The frozen liver was homogenized and purified in TRIzol (Thermo Fisher Scientific) or RNeasy mini kit (Qiagen). SuperScript complementary DNA synthesis kit (Quantabio) was used. Quantitative reverse transcription-PCR using PerfeCTa SYBR Green Fast Mix (Quantabio) was performed in triplicate on the Roche LightCycler 480. Gene expression was normalized to *Ywhaz* or *PPIA* using a comparative threshold cycle (ΔΔCt). See Supplemental Table S4, http://links.lww.com/HC9/B935 for primer sequences.

### Primary mouse hepatocyte isolation

Primary mouse hepatocytes (PMHs) were isolated from wild-type C57BL/6J male mice aged over 12 weeks via two-step collagenase perfusion, as previously described.^30^ Hepatocytes were purified using Percoll (Cytiva, 17089109) gradient centrifugation and plated in HPM on rat tail type-1 collagen-coated plates (Thermo Fisher Scientific, CB-40236).

### *In vitro* MASH conditioning with free fatty acids and fructose

PMH and THLE-5B human hepatocytes were maintained in DMEM with 10% fetal bovine serum, 2 mM l-glutamine, and 1× penicillin/streptomycin, and routinely tested for mycoplasma (MilliporeSigma, MP0025). Oleic acid (OA; MilliporeSigma, O1008) was prepared in DMEM with 1% lipid-free bovine serum albumin (MilliporeSigma, A8806). Palmitic acid (PA; MilliporeSigma, P0500) was prepared in 100% ethanol and 1% lipid-free bovine serum albumin, warmed to 55 °C and sonicated at room temperature (RT). OA+PA were filter sterilized and prepared at a 2:1 ratio—750µM for THLE-5B cells and 400µM for PMH in 0.2% ethanol, which was used as vehicle control. Fructose (Sigma, F1027) was dissolved in DMEM and filtered to 100 mM for THLE-5B and 50 mM for PMH. Hepatocytes underwent OA+PA +/− fructose conditioning for 48–72 hours. Media was changed at 24 hours for 72 hours conditioning.

### KHK overexpression

THLE-5B cells were transfected using Lipofectamine 3000 with human KHK (pcDNA3.1-C-(k)DYK-KHK, GeneScript, OHu18645) or pcDNA3.1-C-(k)DYK empty vector control, and treated with vehicle, free fatty acids (FFA), fructose, and mannose 8 hours after transfection. Cells were collected 48 hours after treatment.

### Mannose and galactose *in vitro* treatment protocol

Mannose or galactose was dissolved in either PBS or DMEM to 10 and 25 mM, filter sterilized and given for 48–72 hours.

### ORO staining and quantification

ORO staining was done after cell fixation with 10% formalin at RT ×1 hour and 60% isopropanol ×5 minutes. Washes were done with double-distilled water. Images were obtained, and ORO was eluted with 100% isopropanol. Optical density (OD) was measured at 500 nm using 100% isopropanol as blank. Methylene blue was destained (40% methanol and 4% acetic acid). OD was taken at 665 nm using 40% methanol and 4% acetic acid as blank. For normalization, ORO OD was divided by corresponding methylene blue OD.

### Western blot

Antibodies were used as indicated in Supplemental Table S5, http://links.lww.com/HC9/B935.

### Immunofluorescence

Cells were fixed with 4% paraformaldehyde in PBS at RT. Cells were blocked with 0.1% bovine serum albumin in PBS with 0.2% saponin and 0.02% sodium azide, labeled with anti-KHK and secondary antibody at RT (Supplemental Table S5, http://links.lww.com/HC9/B935), stained with DAPI (MP Biomedicals, 157574), mounted using Vectashield (Vector laboratories, H-1000), and imaged on a Leica DMi8. Fluorescence was analyzed using ImageJ.

### Human precision-cut liver slices

Human precision-cut liver slices (hPCLS) were generated as previously described^31^ and in accordance with the Institutional Review Board (IRB) at Mount Sinai and both the Declarations of Helsinki and Istanbul. Briefly, remnants of de-identified, surgically resected explanted human livers were used. Liver slices were prepared at 200 µm thickness. The slices were restored with 24-hour incubation in William E GlutaMAX media (Thermo Fisher Scientific) supplemented with 25 mM glucose and 50 µg/mL gentamicin (Thermo Fisher Scientific). After restoration, liver slices were treated with PBS and 1 or 25 mM mannose for 24 and 48 hours. Total mRNA was extracted for reverse transcription-quantitative PCR. Culture media was collected for secreted COL1A1^31^ and albumin^32^ as previously described. Immunofluorescence staining was done as previously described^31^ and analyzed using ImageJ.

### Statistics

GraphPad Prism software (version 9.1.1; GraphPad Software Inc.) was used. Data are expressed as mean ± SD. Differences between groups were analyzed by two-tailed, unpaired Student *t* test or one-way ANOVA, followed by Dunnet or Šidák post hoc correction. Fold changes were analyzed by one-sample *t* test against the theoretical mean of 1. *p*<0.05 was considered statistically significant.

## RESULTS

### Mannose prevents and treats steatosis in the FAT-MASH murine model

To examine mannose as a potential therapy for MASH *in vivo*, we used the FAT-MASH mouse model.^4^^,^^25^ Eight-week-old male C57BL/6J mice were given a high fat, cholesterol, and sugar diet with low-dose CCl_4_ for 12 weeks and placed on either a low (5% weight/volume) or high (20% weight/volume) dose of mannose in the drinking water ad libitum (Figure 1B). Mannose supplements were initiated at either the start of the protocol (“prevention”) or 6 weeks into the 12-week FAT-MASH regimen when steatosis and fibrosis are already present (“therapy”). All mice gained weight similarly, regardless of diet or mannose dosing group, and food intake was comparable for all FAT-MASH mice (Supplemental Figures S1A and B, http://links.lww.com/HC9/B935). Despite reduced water intake in the mannose prevention group, the mannose consumed was comparable to the therapy group at each dose (Supplemental Figures S1C and D, http://links.lww.com/HC9/B935).

Consistent with previous reports,^25^^,^^33^ FAT-MASH mice had histological evidence of MASH at 6 and 12 weeks, including hepatic steatosis with elevated liver TG, liver cholesterol, liver weight, and liver/body weight ratio compared to normal diet (ND) (Figures 1C and D and Supplemental Figures S1E–G, http://links.lww.com/HC9/B935). Serum ALT, AST, and bilirubin were analyzed, but only AST demonstrated an increase in 12-week FAT-MASH (Supplemental Figures S1H–K, http://links.lww.com/HC9/B935).

Mannose supplementation had a dose-dependent effect on alleviating steatosis with reduced size and number of lipid vesicles in both the prevention and therapy groups (Figure 1C). High-dose mannose reduced liver TG (Figure 1D), liver weight, and liver/body weight ratios (Supplemental Figures S1E and F, http://links.lww.com/HC9/B935) in both prevention and therapy groups. In the therapeutic groups, high-dose mannose decreased liver cholesterol while low-dose improved serum AST levels (Supplemental Figures S1G and I, http://links.lww.com/HC9/B935).

To quantify liver steatosis, we applied an automated, high-resolution digital pathology and artificial intelligence–based platform (FibroNest) to quantify multiple fibrotic and macrosteatotic phenotypic traits.^4^^,^^28^^,^^34^^,^^35^ FibroNest measurements confirmed that the FAT-MASH regimen induced steatosis by 6 weeks and peaked at 12 weeks (Figure 1E). In 12-week FAT-MASH livers, there were significant increases in macrosteatosis area from 0.3% (ND) to 24% and fat vacuole counts (Figures 1F and G and Supplemental Table S1, http://links.lww.com/HC9/B935). Mannose reduced steatotic area and normalized fat vacuole counts in a dose-dependent manner (Figures 1E–G). Low-dose and high-dose mannose treatments in the prevention group reduced steatosis by 26% and 60%, respectively. Similar reductions were seen in the therapy group; low-dose and high-dose mannose treatments reduced steatosis by 43% and 70%, respectively (Supplemental Table S1, http://links.lww.com/HC9/B935).

Together, these data demonstrate that oral mannose lowers liver steatosis with reduced hepatic TG and cholesterol accumulation in the FAT-MASH mouse model.

### Mannose dampens fructose metabolism and fructose-mediated steatosis via KHK

**FIGURE 2:**
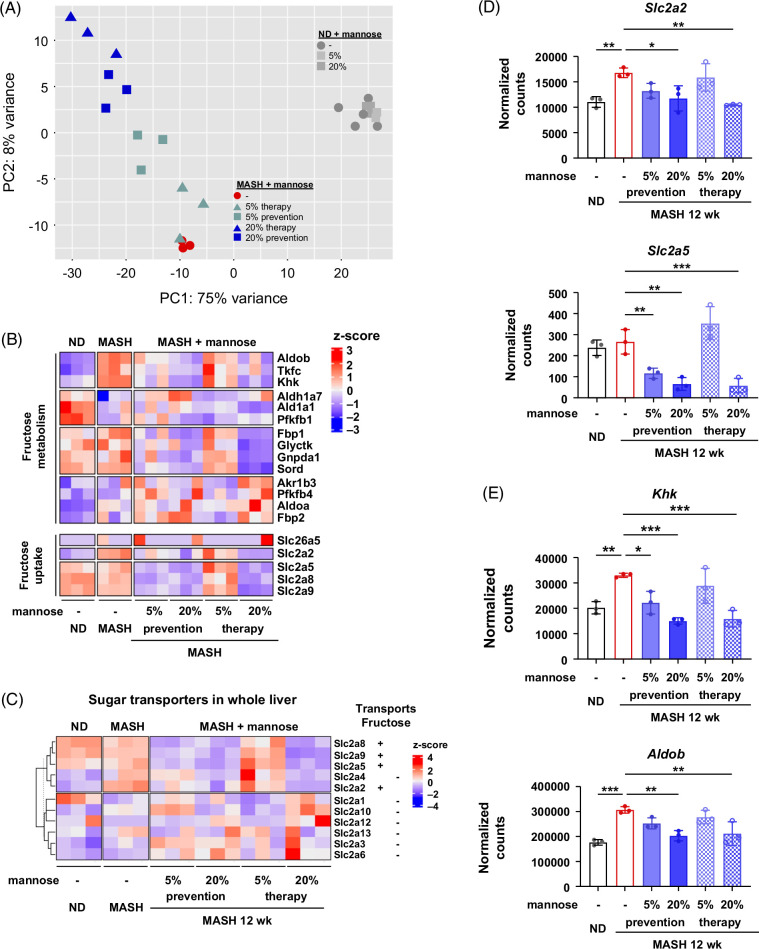
Mannose induces transcriptional changes in fructose metabolism and uptake genes. (A) Principal component analysis plot of differentially expressed genes identified by bulk RNA sequencing of whole livers from all experimental groups (n=3–5 per group). (B) Heatmap showing normalized z-scores for expression of genes involved in fructose metabolism and uptake. (C) Heatmap showing normalized z-scores for expression of genes involved in transmembrane sugar transport (*Slc2a* family). Reported fructose transporters are shown. (D) Bar plots showing normalized expression of fructose transporters *Slc2a2* encoding Glut2 and *Slc2a5* encoding Glut5. (E) Bar plots showing normalized expression of *Khk* and *Aldob.* Statistical comparisons are by one-way ANOVA with Dunnett post hoc test for multiple comparisons (*p<0.05, **p<0.01, and ***p<0.001). Abbreviations: KHK, ketohexokinase; MASH, metabolic dysfunction–associated steatohepatitis; ND, normal diet.

To explore the mechanisms of mannose on MASH steatosis, we performed bulk RNA-seq on mouse livers. Across all treatment groups, 83% of the transcriptional variation was captured in the first 2 principal components. Mannose induced substantial changes in a dose-dependent manner in FAT-MASH mice (Figure 2A). Gene set enrichment analysis showed mannose downregulated adipogenesis and fatty acid metabolism and upregulated cholesterol homeostasis (Supplemental Figures S2 and S3, http://links.lww.com/HC9/B935).

Given the known roles of fructose in MASH^36^^–^^38^ and the metabolic intersection with mannose (Figure 1A), we hypothesized that mannose exerts antisteatotic effects through fructose metabolism. Analysis of RNA-seq data revealed fructose uptake and metabolism genes to be significantly changed with mannose treatments (Figure 2B). The aforementioned list of genes was obtained from GO_BP terms “fructose metabolic process” and “fructose membrane transport.” Mannose reduced the expression of fructose transporters, which are members of the SLC2A (GLUT) family of facilitative hexose transporters (Figure 2C). No changes were seen in the non-fructose SLC2A members (Figure 2C). We specifically queried *Slc2a2* and *Slc2a5*, which are fructose transporters associated with MASLD,^39^ and found that mannose significantly dampened their expressions (Figure 2D). In addition, mannose-attenuated genes are involved in fructose metabolism, particularly *Khk* and *Aldob*, the first 2 steps of fructolysis (Figures 2B and E).

**FIGURE 3:**
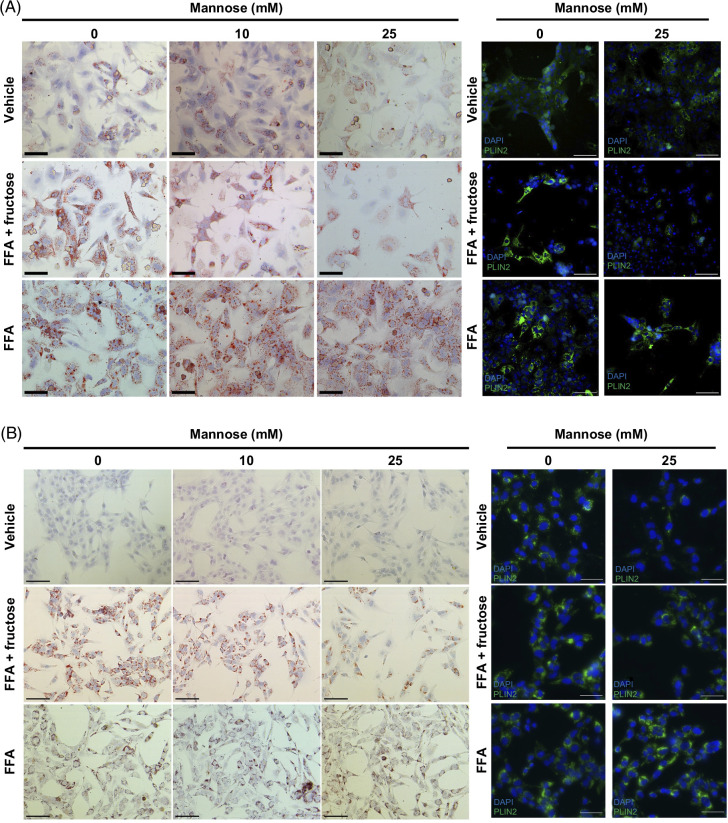
Mannose reduces steatosis *in vitro* and is fructose-mediated. Representative photomicrographs of ORO-stained and PLIN2 immunofluorescence staining of primary mouse hepatocytes (A) or THLE-5B human hepatocytes (B). Scale bar: 100 µm. Abbreviations: FFA, free fatty acids; ORO, Oil Red O.

Given that mannose dampened fructose metabolism pathways in FAT-MASH livers, we next tested whether the antisteatotic effects of mannose were dependent on the presence of fructose. To mimic FAT-MASH conditions in PMHs and the human hepatocyte cell line THLE-5B, we used a treatment protocol containing FFA and fructose.^40^^,^^41^ Incubation of hepatocytes with OA+PA (2:1 cocktail) and fructose for 48–72 hours induced steatosis (Figure 3). Similar to findings in the whole liver, mannose dampened steatosis in PMHs, with a 30% reduction in steatosis with 25 mM mannose treatment (Figure 3A and Supplemental Figure S4A, http://links.lww.com/HC9/B935). However, steatosis was refractory to mannose therapy in PMHs exposed to FFA alone (Figure 3A and Supplemental Figure S4A, http://links.lww.com/HC9/B935). These findings were confirmed in human hepatocytes, where mannose reduced steatosis by 33%, but only when fructose was present (Figure 3B and Supplemental Figure S4B, http://links.lww.com/HC9/B935). These effects were specific to mannose, as galactose (an epimer of mannose) failed to significantly improve steatosis in any MASH condition (Supplemental Figure S5, http://links.lww.com/HC9/B935).

**FIGURE 4:**
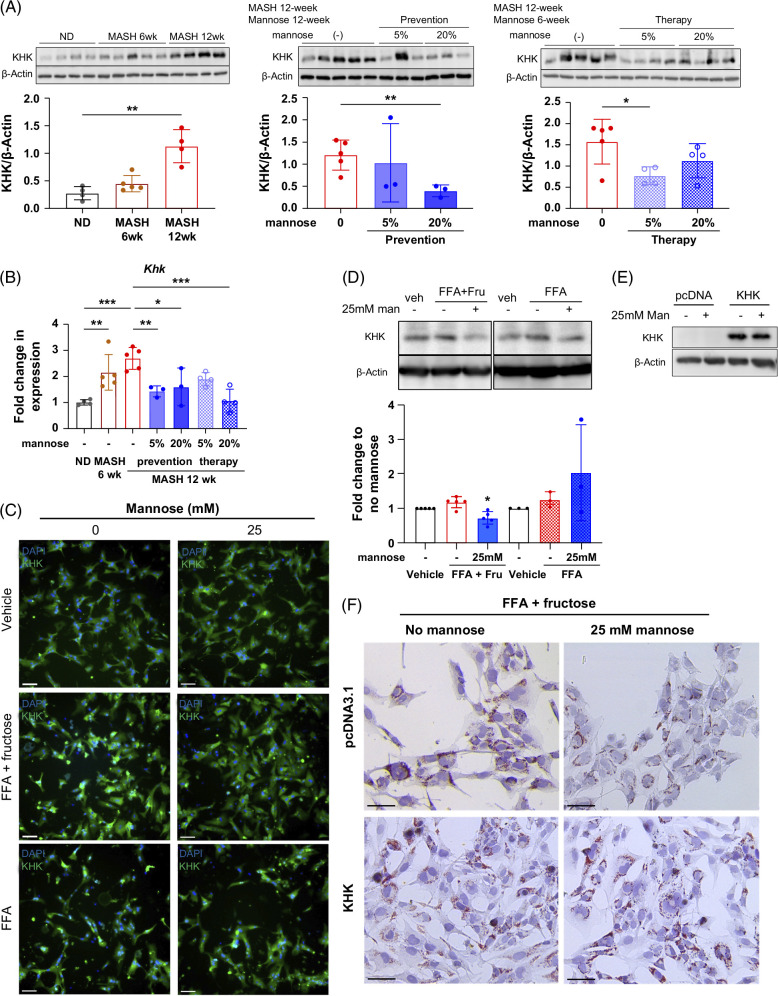
Mannose treatment decreases KHK *in vivo* and *in vitro*, and KHK overexpression abrogates mannose’s benefit of reducing steatosis. (A) Western blot of KHK in whole liver lysates, with β-actin used for loading control. Semiquantitative densitometry (ImageJ) of KHK/β-actin ratios is shown. (B) Validation of *Khk* expression by qPCR from FAT-MASH mouse livers; n=3–5. (C) Immunofluorescent staining of KHK (green) ﻿with DAPI (blue) counterstaining in PMHs. Cells are conditioned with FFA + fructose, FFA alone, or vehicle, ± mannose treatment, for 72 hours. Scale bar: 100 µm. (D) Representative western blot of KHK in THLE-5B cells conditioned with FFA + fructose (FFA+Fru), FFA alone, or vehicle, ± mannose treatment. β-Actin is used as a loading control. Bar chart (below) showing semiquantitative densitometry (ImageJ) of KHK/β-actin normalized expression. Fold changes to no mannose treatments for each condition are shown (n=3–5) with one sample t-test for statistical comparison (* *p*<0.05). (E) Representative western blot showing overexpression of KHK or empty vector control (pcDNA3.1) in THLE-5B cells, conditioned with FFA + fructose, ± mannose treatment for 48 hours. β-Actin is used for loading control. (F) ORO staining of THLE-5B cells expressing endogenous KHK or empty vector (pcDNA3.1) conditioned with FFA + fructose, ± mannose treatment. Scale bar: 50 µm. Statistical comparisons are by one-way ANOVA with Dunnett post hoc test for multiple comparisons, Student *t* test, or one-sample *t* test (*p<0.05, **p<0.01, and ***p<0.001). Abbreviations: FAT-MASH, fat-and-tumor MASH; FFA, free fatty acids; KHK, ketohexokinase; MASH, metabolic dysfunction–associated steatohepatitis; ND, normal diet; ORO, Oil Red O; PMHs, primary mouse hepatocytes; qPCR, quantitative PCR.

We next focused on KHK, the rate-limiting step of fructose metabolism (Figure 1A). KHK promotes MASH in murine models and humans.^13^^–^^16^^,^^42^ We found that KHK was increased in 12-week MASH murine livers compared to ND. Mannose reduced KHK expression in all supplemented groups (Figures 4A and B). *In vitro*, mannose dampened the elevated KHK induced by FFA + fructose conditioning in both PMHs and human hepatocytes (Figures 4C and D). KHK levels were also refractory to mannose when conditioned by FFA alone (Figures 4C and D). If mannose attenuates steatosis through reducing KHK, we hypothesized that increasing KHK levels would negate the effects of mannose. To test this, we overexpressed KHK in human hepatocytes following FFA + fructose and mannose treatments (Figure 4E), and, as predicted, KHK overexpression abrogated the antisteatotic effect of mannose (Figure 4F). Taken together, these data demonstrate a therapeutic role for mannose in fructose-induced hepatic steatosis by dampening KHK expression.

### Mannose improves fibrosis in FAT-MASH and CCl_4_ murine models

**FIGURE 5:**
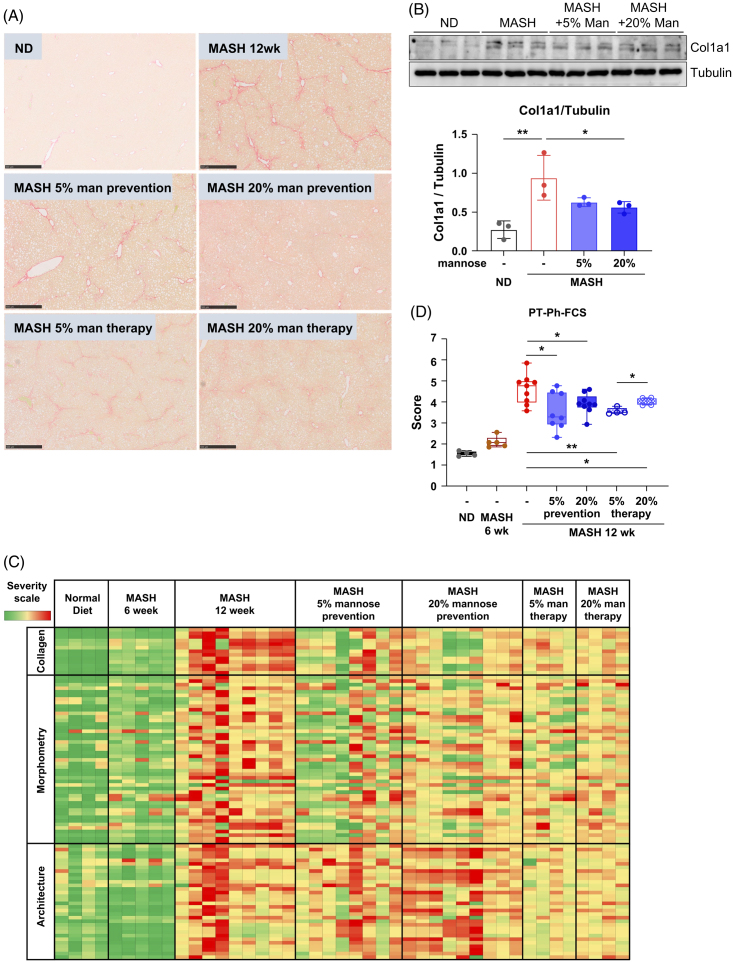
Mannose attenuates fibrosis in FAT-MASH mice. (A) Representative images of Sirius Red staining for collagen from whole liver sections. Scale bar: 500 µm. (B) Western blot of Col1a1 in whole liver lysates from ND or MASH mice without or with mannose supplements. Tubulin is used as a loading control, and Col1a1/tubulin ratios are quantified with ImageJ, and compared by one-way ANOVA with Dunnett post hoc test for multiple comparisons (*p<0.05, **p<0.01). (C) Quantitative, phenotypic digital pathology and AI-based quantification of Sirius red collagen staining. (D) Principal quantitative fibrosis traits are aggregated into a Phenotypic Fibrosis Composite Score, normalized to the parenchymal (nonsteatotic) tissue present. n=4–9 mice per group. Results expressed as mean ± SD and compared by Student *t* test (*p<0.05 and **p<0.01). Abbreviations: FAT-MASH, fat-and-tumor MASH; MASH, metabolic dysfunction–associated steatohepatitis; ND, normal diet; PT-Ph-FCS, Phenotypic Fibrosis Composite Score.

Fibrosis severity is the primary driver of patient outcomes in MASH.^43^ HSCs are the major cell type responsible for liver fibrogenesis. We have previously shown that mannose dampens HSC activation *in vitro*,^23^ and sought to examine the antifibrotic effects of mannose supplementation in MASH-fibrosis. Consistent with previous reports,^4^^,^^25^^,^^33^ mice fed the FAT-MASH diet developed substantial bridging fibrosis by 12 weeks (Figure 5A). Importantly, low-dose and high-dose mannose supplementation effectively mitigated liver fibrosis in both prevention and therapy groups (Figure 5A). Western blot on liver lysates confirmed increased collagen (COL1A1) in FAT-MASH livers when compared to ND, which decreased with mannose supplementation (Figure 5B).

To obtain an unbiased and comprehensive analysis of fibrosis, we utilized FibroNest, a quantitative digital pathology and artificial intelligence platform. FibroNest measured 3 fibrosis subphenotypes: (1) amount of collagen deposition, (2) fiber morphometry, and (3) fibrosis architecture.^28^ The principal quantitative fibrosis traits for these fibrosis subphenotypes are displayed in a phenotypic heatmap, with the ND livers having the least fibrosis and the MASH livers with the most at 12 weeks. Importantly, fibrosis severity was reduced in every mannose treatment group (Figure 5C). In a 2D fibrosis chart of histological fibrosis traits, prevention and therapy mannose groups restored the collagen profile closer to the ND group (Supplemental Figure S6, http://links.lww.com/HC9/B935). Similarly, all groups receiving mannose treatment showed a significant reduction in Phenotypic Fibrosis Composite Score as compared to the MASH (12 wk) (Figure 5D and Supplemental Table S2, http://links.lww.com/HC9/B935).

**FIGURE 6:**
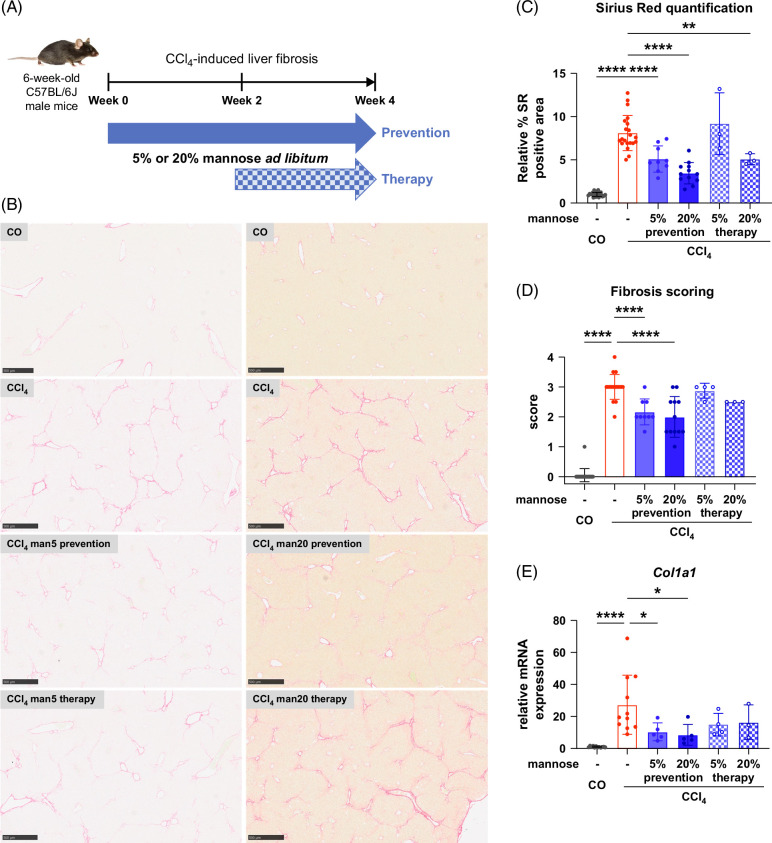
Mannose attenuates hepatic fibrosis in a CCl_4_ murine model. (A) Graphical outline of the CCl_4_-induced liver fibrosis regimen. (B) Representative images of Sirius Red staining from whole liver sections. Scale bar: 500 µm. (C) Sirius Red quantification and (D) fibrosis scoring from histology images; n=3–21. (E) Quantitative PCR analysis of *Col1a1* mRNA expression; n=3–12. Results expressed as mean ± SD and compared by Student *t* test (*p<0.05, **p<0.01, ***p<0.001, and ****p<0.0001).

Given that mannose directly mitigates HSC activation,^23^ we hypothesized that mannose exerts therapeutic antifibrotic effects not only in MASH but in other liver injury models. To test this, we used the chemotoxic CCl_4_ mouse model^26^ to induce fibrosis. We supplemented low (5%) or high (20%) dose mannose in drinking water, commencing at either the start (prevention) or 2-week CCl_4_ timepoint (therapy) (Figure 6A). At 4 weeks of CCl_4_ injury, mice developed substantial fibrosis without steatosis (Figure 6B). Mannose effectively reduced CCl_4_-induced fibrosis in both prevention and therapy groups, especially with high doses (Figure 6C). Mannose also lowered fibrosis scoring and *Col1a1* expression, particularly in the prevention group (Figures 6D and E). Here, we uncover mannose as a novel therapeutic that can mitigate liver fibrosis across 2 distinct *in vivo* mouse models.

### Mannose reduces KHK and serves antifibrotic roles in cirrhotic human explanted liver slices

To test the therapeutic relevance of mannose supplementation in MASH patients, we utilized hPCLS, an *ex vivo*, 3-dimensional cell culture model generated from patients’ explanted livers at the time of transplantation. hPCLS preserves the native architecture and cell types to capture endogenous cell–cell interaction, making it ideal for drug screening with different dosing and timing regimens.^31^^,^^44^ Three patient samples were collected—2 patients with MASH-induced cirrhosis (MASH #1 and #2) and the third with hepatitis C virus-induced cirrhosis (HCV). All three patients were male, age-matched, and had fibrosis stage F4 on the METAVIR scoring system (Supplemental Table S3, http://links.lww.com/HC9/B935). After a 24-hour equilibration period, hPCLS were incubated with mannose (1 or 25 mM) or PBS (vehicle control) for 24 and 48 hours. Hepatocytes remained viable during mannose treatments with stable albumin secretion (Supplemental Figure S7, http://links.lww.com/HC9/B935).

**FIGURE 7:**
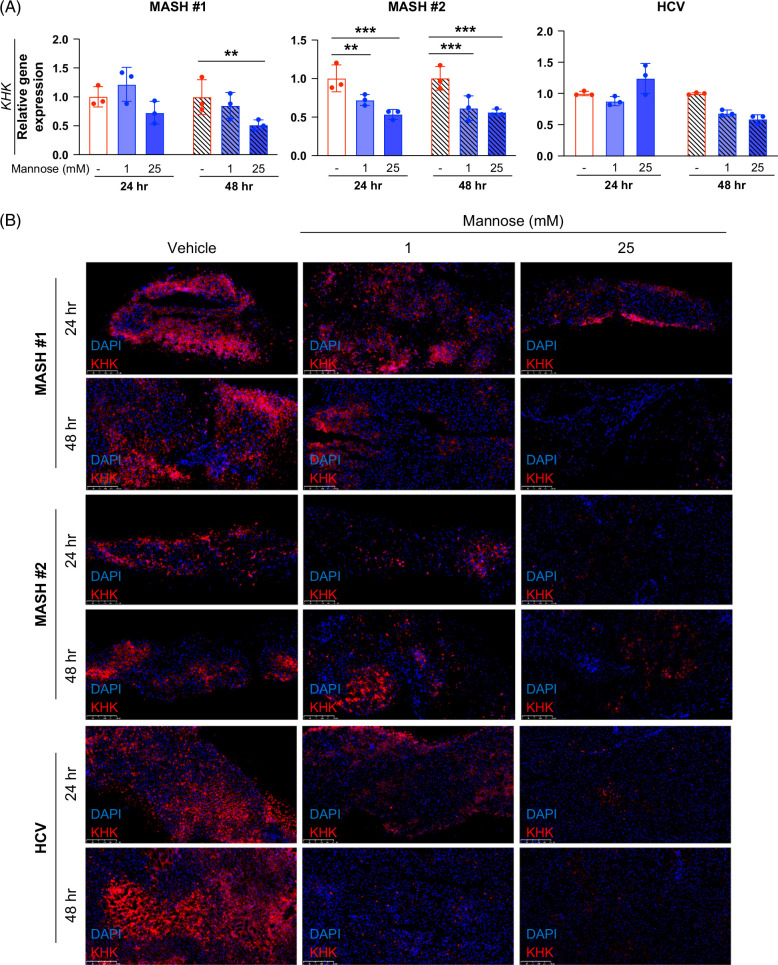
Mannose decreases KHK expression in human MASH precision-cut liver slices. (A) Quantitative PCR of *KHK* mRNA expression. (B) Immunofluorescent staining for KHK (red), counterstained with DAPI (blue). Scale bar: 250 µm. Statistical comparisons are by one-sample *t* test and two-way ANOVA with Dunnett post hoc test for multiple comparisons (**p<0.01 and ***p<0.001). Abbreviations: KHK, ketohexokinase; MASH, metabolic dysfunction–associated steatohepatitis.

**FIGURE 8:**
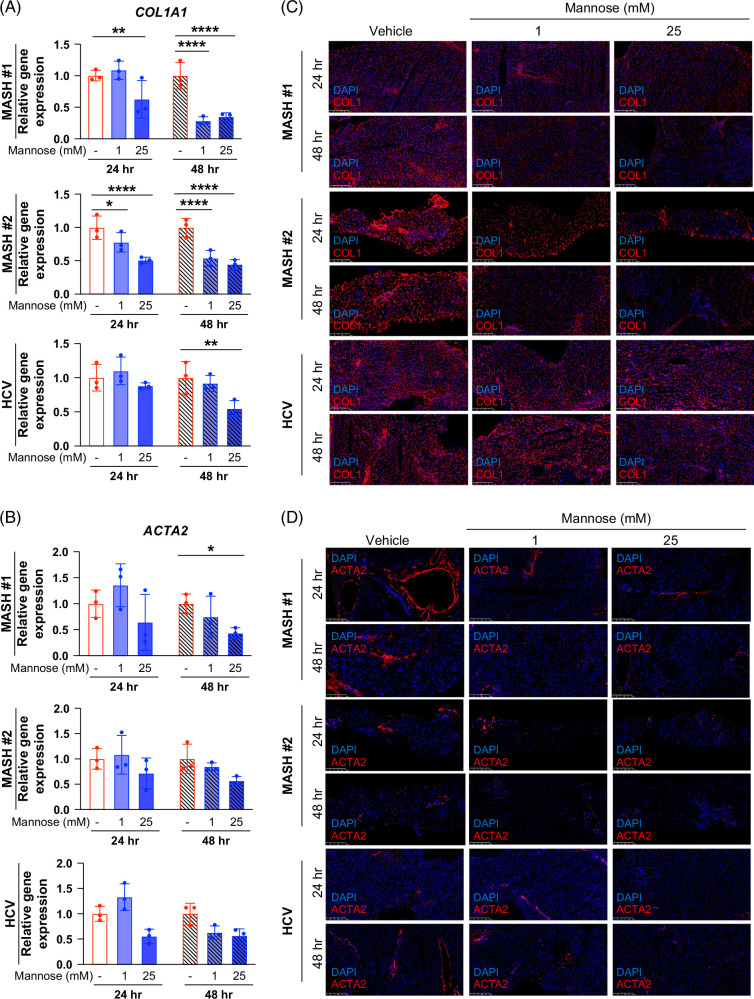
Mannose reduces fibrosis and dampens fibrogenic gene expression in human MASH precision-cut liver slices. Quantitative PCR of *COL1A1* (A) and *ACTA2* (B) mRNA expressions. Results are presented as fold change to vehicle control in each duration group. (C) Immunofluorescent staining for COL1 (red), counterstained with DAPI (blue) with bar charts for quantification, normalized by the number of nuclei. Scale bar: 250 µm. (D) Immunofluorescent staining for ACTA2 (red) and counterstaining with DAPI (blue). Scale bar: 250 µm. Statistical comparisons are by one-sample *t* test and two-way ANOVA with Dunnett post hoc test for multiple comparisons (*p<0.05, **p<0.01, and ****p<0.0001). Abbreviation: MASH, metabolic dysfunction–associated steatohepatitis.

Mannose reduced KHK expression in both MASH patient samples with the strongest effects seen with 25 mM and at 48-hour mannose treatments (Figure 7 and Supplemental Figure S8, http://links.lww.com/HC9/B935). For HCV, mannose showed similar effects as MASH specimens (Figure 7 and Supplemental Figure S8, http://links.lww.com/HC9/B935). Mannose decreased fibrogenic gene expression in both MASH patient samples in a dose-dependent and time-dependent manner. qPCR for *COL1A1* and *ACTA2* expression showed the strongest effect with 25 mM mannose and 48-hour treatment (Figures 8A and B). Mannose supplements decreased COL1A and ACTA2 protein levels in MASH patients in a similar manner (Figures 8C and D and Supplemental Figures S9A and B, http://links.lww.com/HC9/B935). In addition, mannose significantly reduced COL1A1 secretion in a dose-dependent manner in both patients (Supplemental Figure S9C, http://links.lww.com/HC9/B935). Interestingly, 25 mM mannose for 48 hours also decreased expression of COL1A1 and ACTA2 in the HCV patient sample (Figure 8 and Supplemental Figures S9A and B, http://links.lww.com/HC9/B935), but dose and time dependencies seen in MASH samples were not evident. Together, these findings demonstrate that mannose alleviates advanced fibrosis in human cirrhotic liver samples and point to the translational therapeutic potential across different liver diseases.

## DISCUSSION

Our study reveals that mannose supplementation has potential therapeutic roles in both MASH-induced steatosis and fibrosis with exciting translational relevance, as this is the first study to demonstrate antifibrotic effects in human MASH livers.

Here, we show that mannose supplementation can prevent and attenuate steatosis, a finding validated by others,^45^^,^^46^ and fibrosis in FAT-MASH mice. Moreover, mannose treatments in MASH hPCLS led to reduced KHK expression and decreased fibrosis. We establish that mannose mitigates steatosis through decreasing KHK and that these effects are dependent on the presence of fructose. The role of mannose as a KHK modulator is appealing; KHK inhibitors have been tested in MASH clinical trials, but mannose may be a simpler way to achieve KHK dampening. It is also possible that mannose reduces steatosis through additional regulations of fructose metabolism, such as fructose transport, microbiome modulation, and inflammatory signaling. Additional studies are needed to examine the effects of mannose on metabolic dysfunction, as well as in cases of MASH that are not fructose-driven.

In this study, our data demonstrate that mannose decreases fibrosis in 2 distinct *in vivo* models, suggesting mannose has antifibrotic effects independent from ameliorating MASH steatosis. Further investigations using single-cell or spatial transcriptomics may delineate cell-specific effects of mannose. However, this offers an exciting positioning of mannose in the landscape of MASH therapeutic development to target both fibrosis and its upstream metabolic injury.

The role of mannose as a novel antifibrotic therapy has the potential to have a high clinical impact as patients with progressive liver fibrosis have limited medical therapies and progress to transplantation.^4^^,^^43^ Our group has previously shown that mannose dampens HSC activation directly *in vivo* and *in vitro*,^23^ and mannose has also been shown to reduce pulmonary and renal fibrosis in murine models.^47^^,^^48^ Our hPCLS findings provide further support for the clinical promise that mannose mitigated fibrosis in liver explants of end-stage MASH patients taken at the time of transplantation. Our murine data also show that mannose can reduce fibrosis in less advanced stages of fibrosis *in vivo*. It is possible that mannose may be clinically effective at earlier fibrosis stages, but further studies are needed. It is interesting to note that mannose showed antifibrotic effects in a patient with HCV-induced fibrosis. This suggests that mannose may be applicable broadly to other end-stage liver diseases and targets HSCs across various disorders. This is supported with mannose-reducing fibrosis in the murine CCl_4_ model, and other *in vivo* models.^23^^,^^24^

The global prevalence of MASH and its rise as a leading indication for liver transplant provides a strong impetus for research efforts to identify effective treatments. Mannose is a simple sugar that is well-tolerated, easily accessible, and already in clinical use for a rare pediatric disease.^49^ Our study is the first to uncover mannose supplementation as an intriguing, potential therapy that can not only improve metabolic dysfunction–associated steatosis but also offers a potential new candidate to treat MASH-fibrosis in mice and humans. We show that mannose can improve MASH by dampening fructose metabolism, which has been shown to be a potent driver of MASH-fibrosis.^11^ Given the safety and tolerability of mannose, its efficacy should be assessed in human MASH, especially in children where no drugs are approved due in part to safety concerns.

## Supplementary Material
