# Supplementary material for: Mannose reduces fructose metabolism and reverses MASH in human liver slices and murine models in vivo
Source: Hepatol Commun. 2025 Mar 21;9(4):e0671. doi: 10.1097/HC9.0000000000000671 (PMC11927666; doi:10.1097/HC9.0000000000000671)
Supplement: Supplementary file 1 [file hc9-9-e0671-s001.pdf]

Supplemental Digital Content - ***Mannose reduces fructose metabolism and reverses MASH in human liver slices and murine models in vivo***, John G. Hong

SDC, Supplemental Methods:

Gene Set Enrichment Analysis (GSEA):

GSEA (Broad Institute, UC San Diego)[1] was performed using the mouse hallmark signature gene sets. Analysis was run as described in the GSEA-MSigDB documentation, using total size-factor-normalized counts (DESeq2) as input, and 1000 permutations, weighted enrichment statistic, and Signal2Noise metric for ranking genes as enrichment parameters. Normalized enrichment scores were compiled and plotted for heatmap visualization using R heatmap package.

SDC, Supplemental reference:

1. Subramanian A, Tamayo P, Mootha VK, et al. Gene set enrichment analysis: a knowledge-based approach for interpreting genome-wide expression profiles. *Proc Natl Acad Sci U S A*. Oct 25 2005;102(43):15545-50. doi:10.1073/pnas.0506580102

SDC, Supplemental Figure S1.

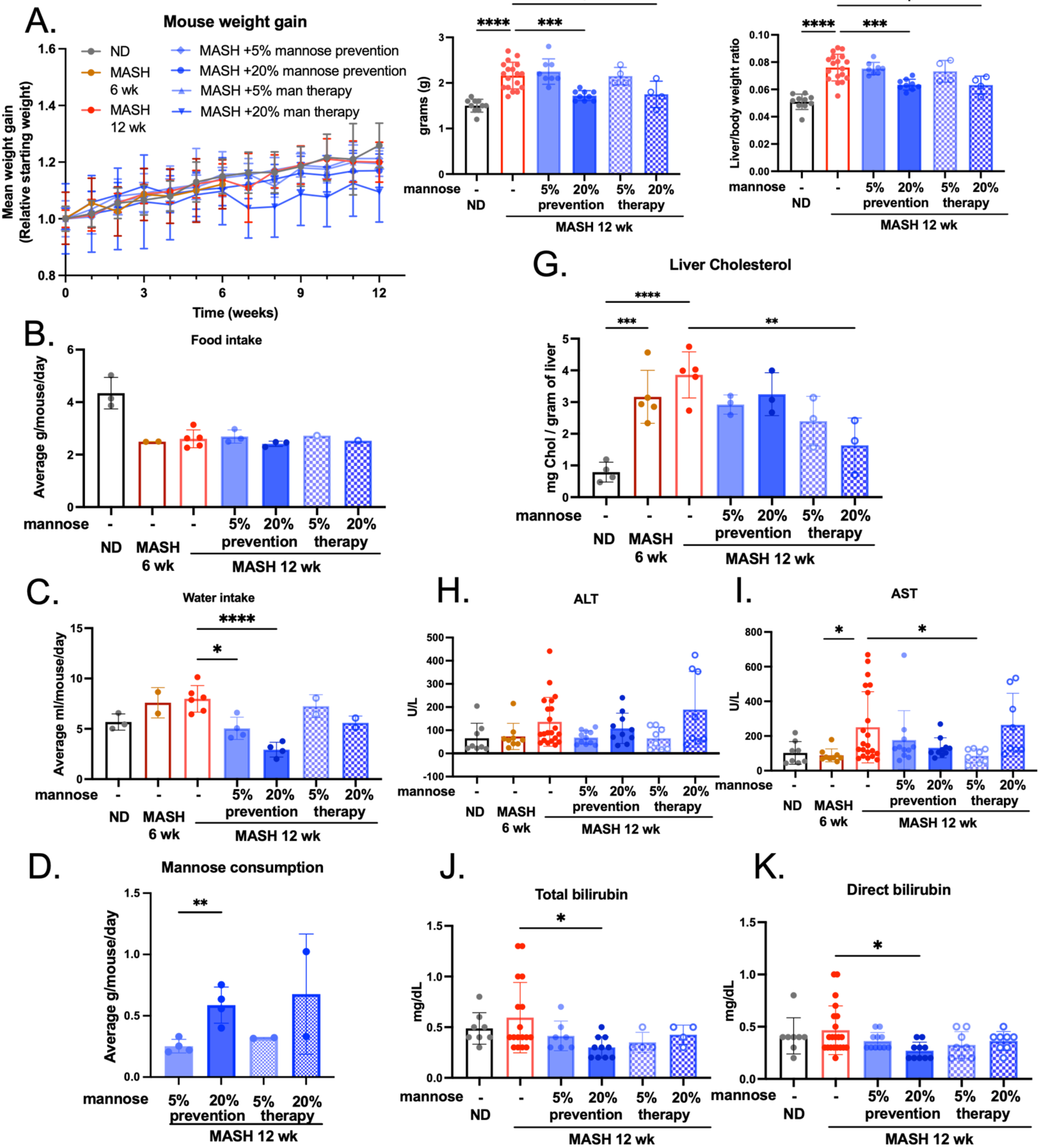

Supplemental Figure S1. Metabolic profile of FAT-MASH mice supplemented with mannose. Mouse total body weight measurements (A), average food intake (B), average water intake (C), and average mannose consumption (D) during course of MASH diet and mannose treatment regimens; n = 1-6 cage per group with 2-5 mice per cage. Liver weight (E), liver to body weight ratio (F), cholesterol levels (G) in whole liver tissue (n=3-5), serum ALT (H), AST (I), total bilirubin (J), and direct bilirubin (K) measured at 12 weeks; n= 4-18 mice per group. Results are expressed as mean ± SD and were compared by Student's t-test or one-way ANOVA (*p<0.05, **p<0.01, ***p<0.001, and ****p<0.0001). ND, normal diet; MASH, FAT-MASH diet; man, mannose.

SDC, Supplemental Figure S2.

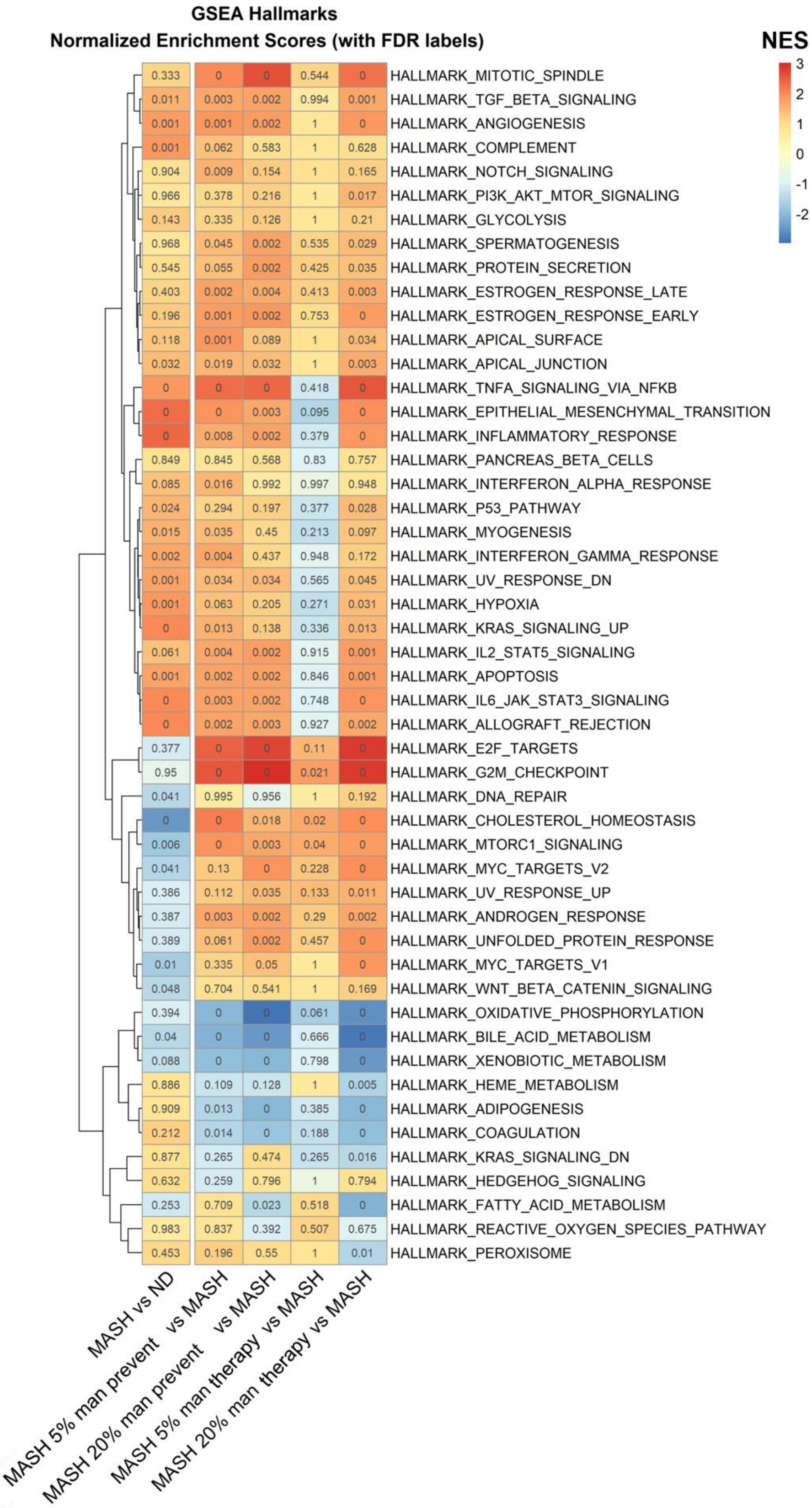

Supplemental Figure S2. Gene Set Enrichment Analysis (GSEA) for Hallmark pathways. Heatmap of compiled results of GSEA from comparative gene expression patterns for different treatment groups of ND, MASH, and MASH + mannose. Hallmark gene set signatures were used for pathway identification. Normalized enrichment scores (NES) are indicated by heatmap color, and false discovery rate (FDR) are labeled within each cell.

SDC, Supplemental Figure S3.

GSEA: Hallmark Cholesterol homeostasis

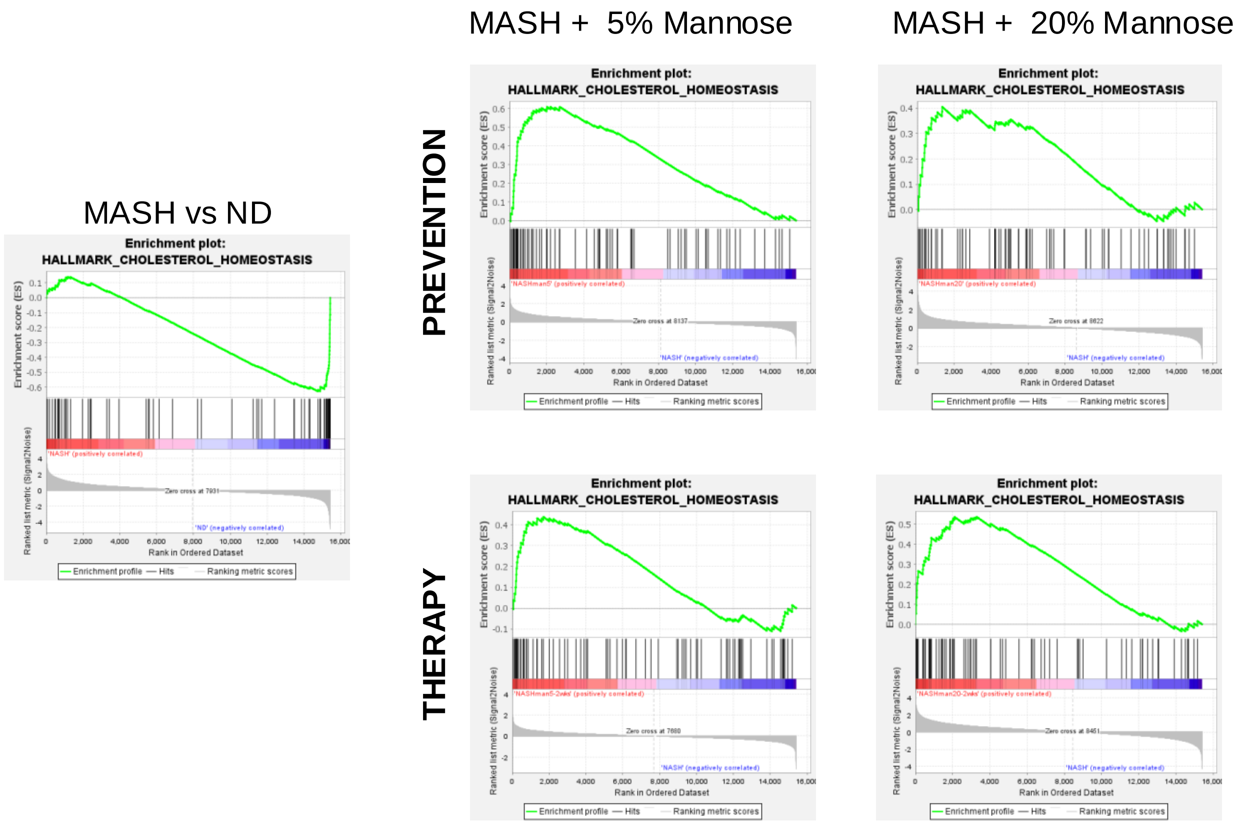

GSEA: Hallmark Adipogenesis

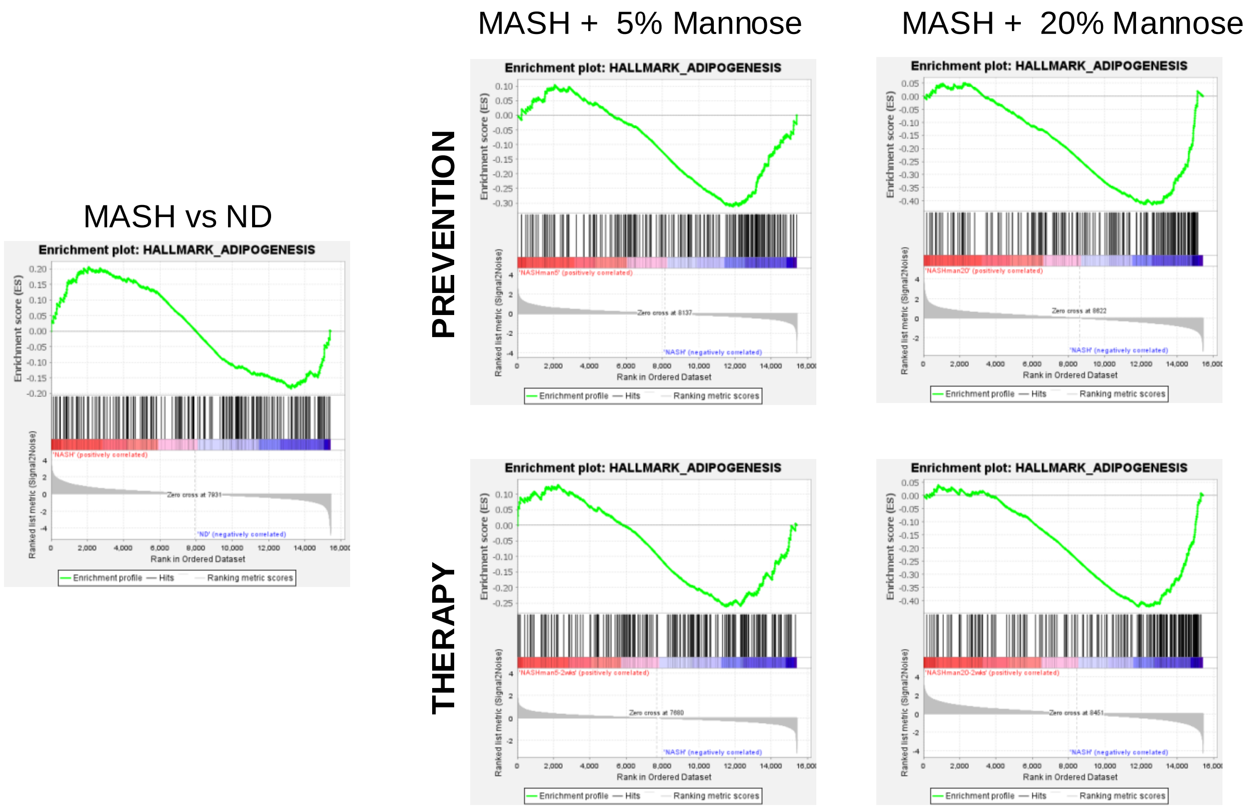

Supplemental Figure S3. Select differentially regulated GSEA Hallmark pathways. Enrichment plots for select hallmark gene sets significantly enriched between indicated treatment groups.

SDC, Supplemental Figure S4.

A.

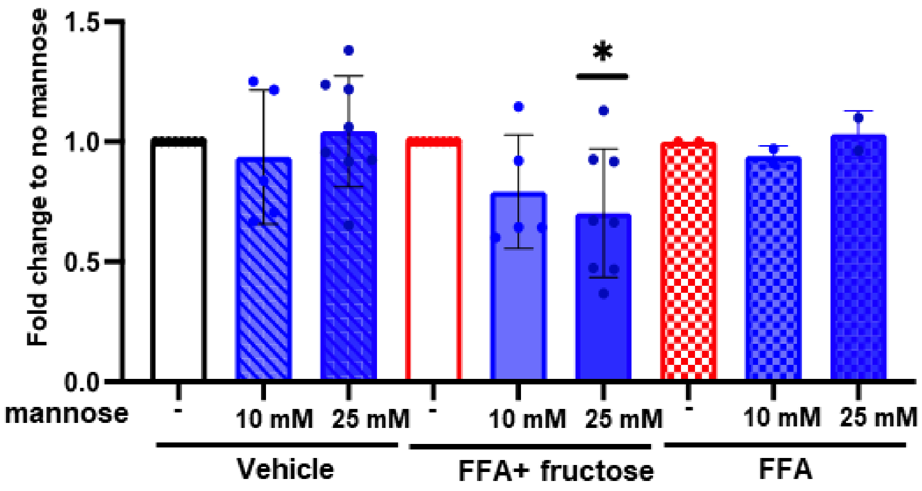

B.

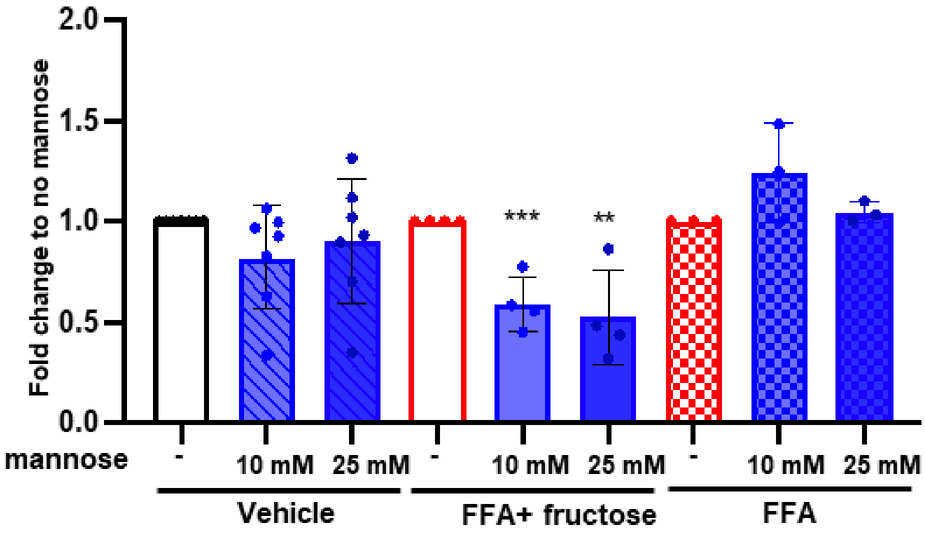

Supplemental Figure S4. ***In vitro*** **Oil Red O quantification.** Oil Red O quantifications in primary mouse hepatocytes (A) and THLE-5B (B). Results are expressed as fold change to no mannose treatments in each condition and analyzed with one sample t-test (*p<0.05, **p<0.01, ***p<0.001).

SDC, Supplemental Figure S5.

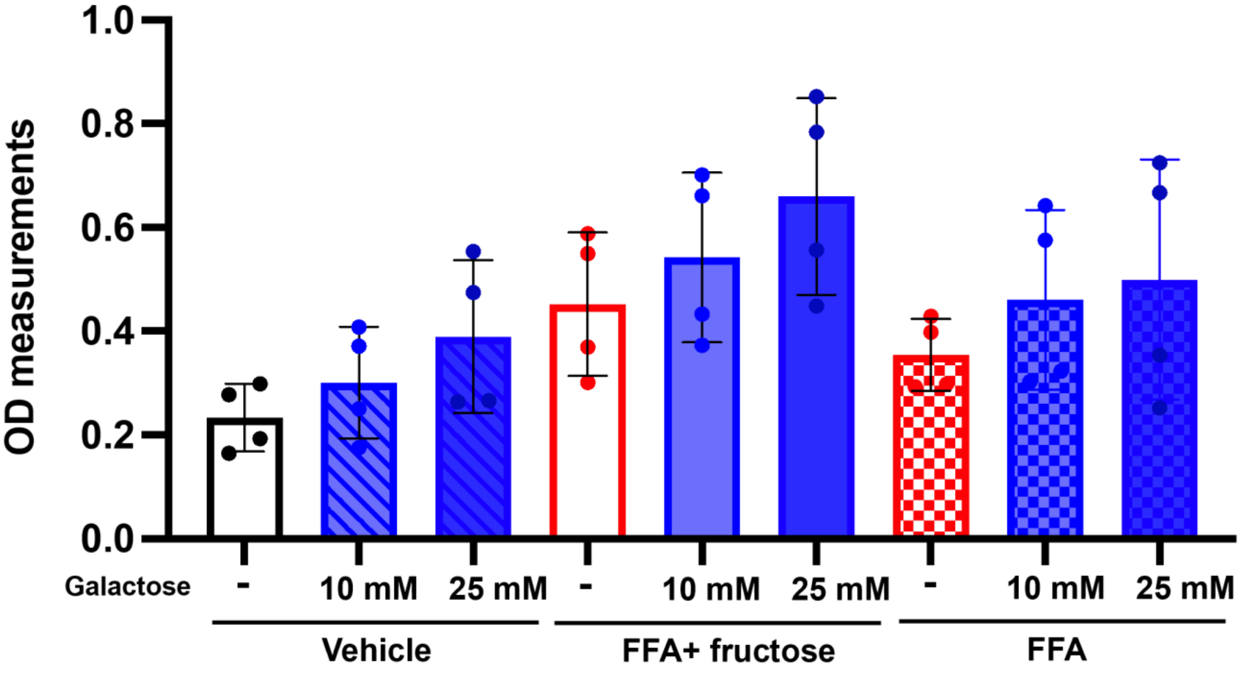

Supplemental Figure S5. *In vitro* galactose treatment. Bar plot showing THLE-5B hepatocytes conditioned with vehicle, FFA + fructose, or FFA alone, without or with 10- or 25-mM galactose for 72 hours (n=4).

SDC, Supplemental Figure S6.

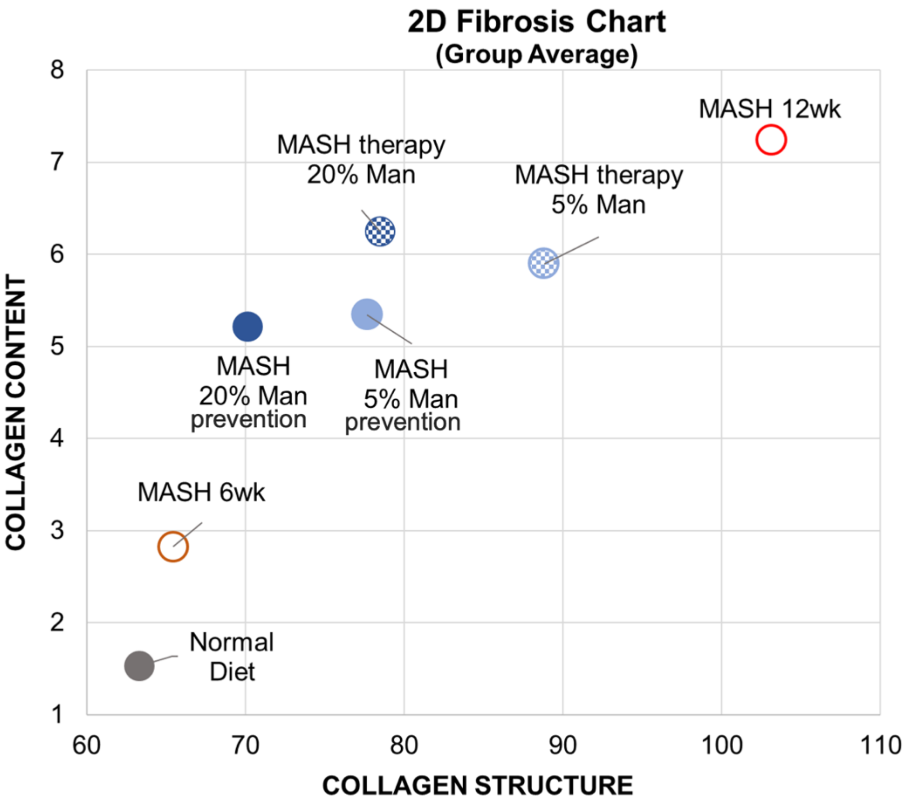

Supplemental Figure S6. Fibrosis profile in FAT-MASH mice. 2D Fibrosis Chart shows the relationship between the collagen deposition and its structure. n=4-9 mice per group.

SDC, Supplemental Figure S7.

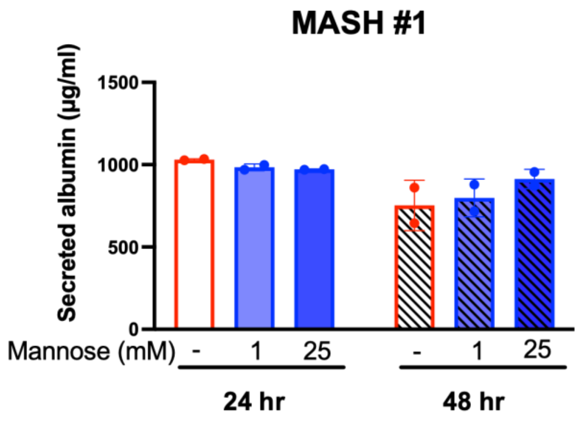

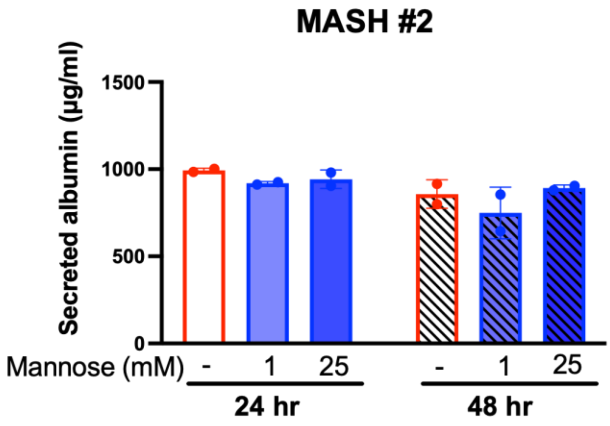

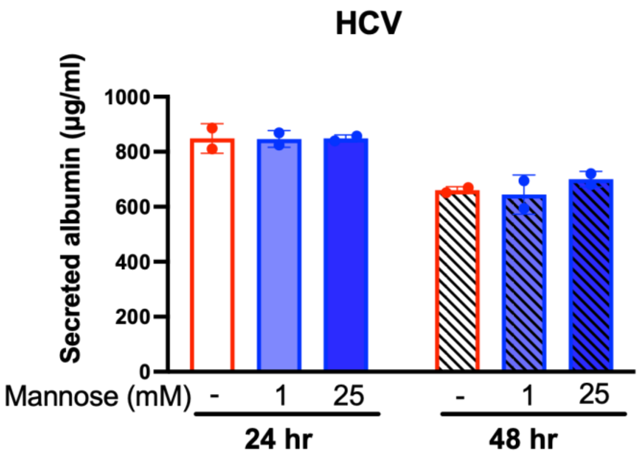

Supplemental Figure S7. Viability profile of human MASH PCLS with mannose treatment. Bar plots showing secreted albumin in human MASH PCLS.

SDC, Supplemental Figure S8.

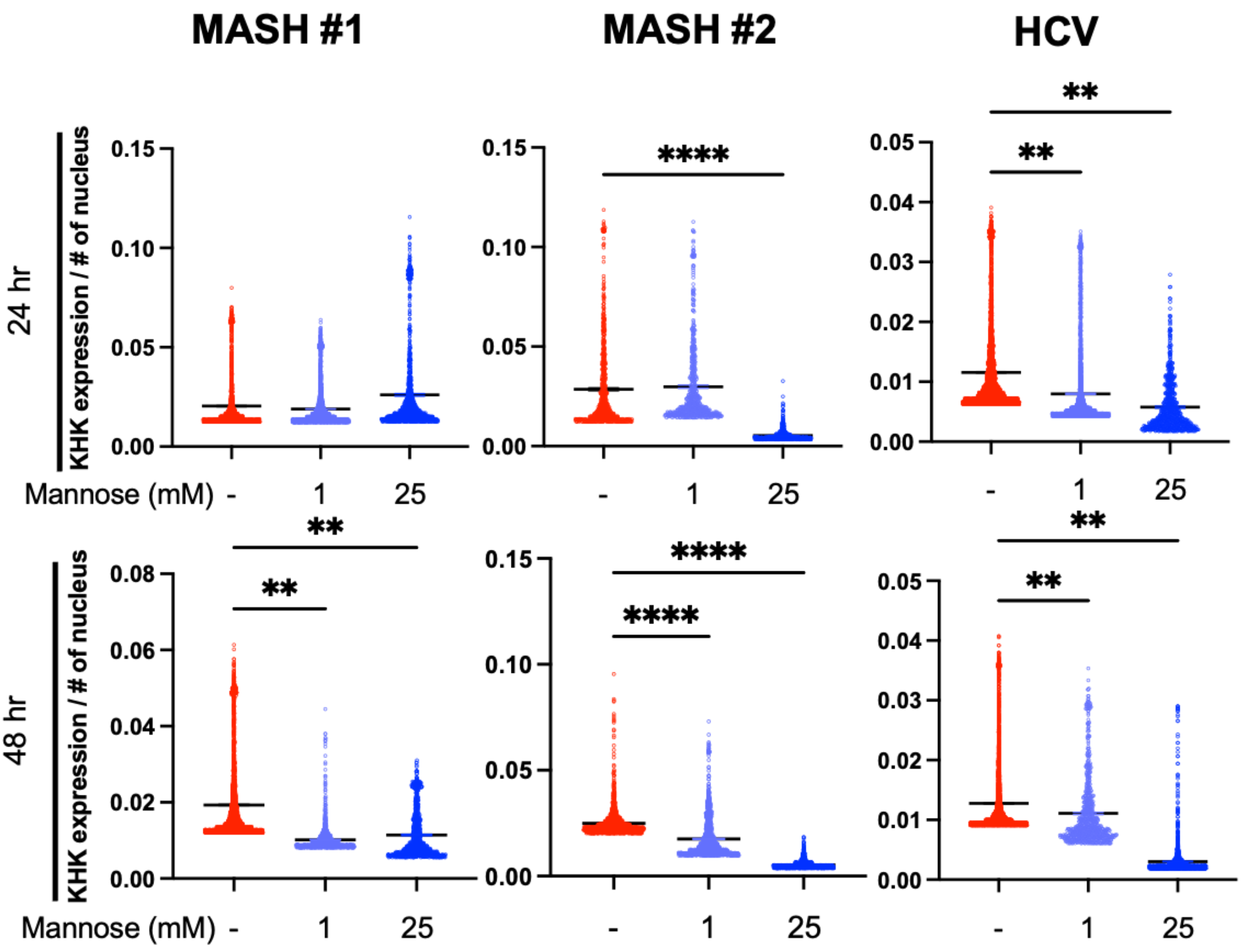

Supplemental Figure S8. KHK expression in human MASH PCLS with mannose treatment. Quantifications of KHK immunofluorescence, normalized by number of nuclei. Statistical comparisons are by one sample t-test and two-way ANOVA with Dunnett's post hoc test for multiple comparisons (**p<0.01, ****p<0.0001).

SDC, Supplemental Figure S9.

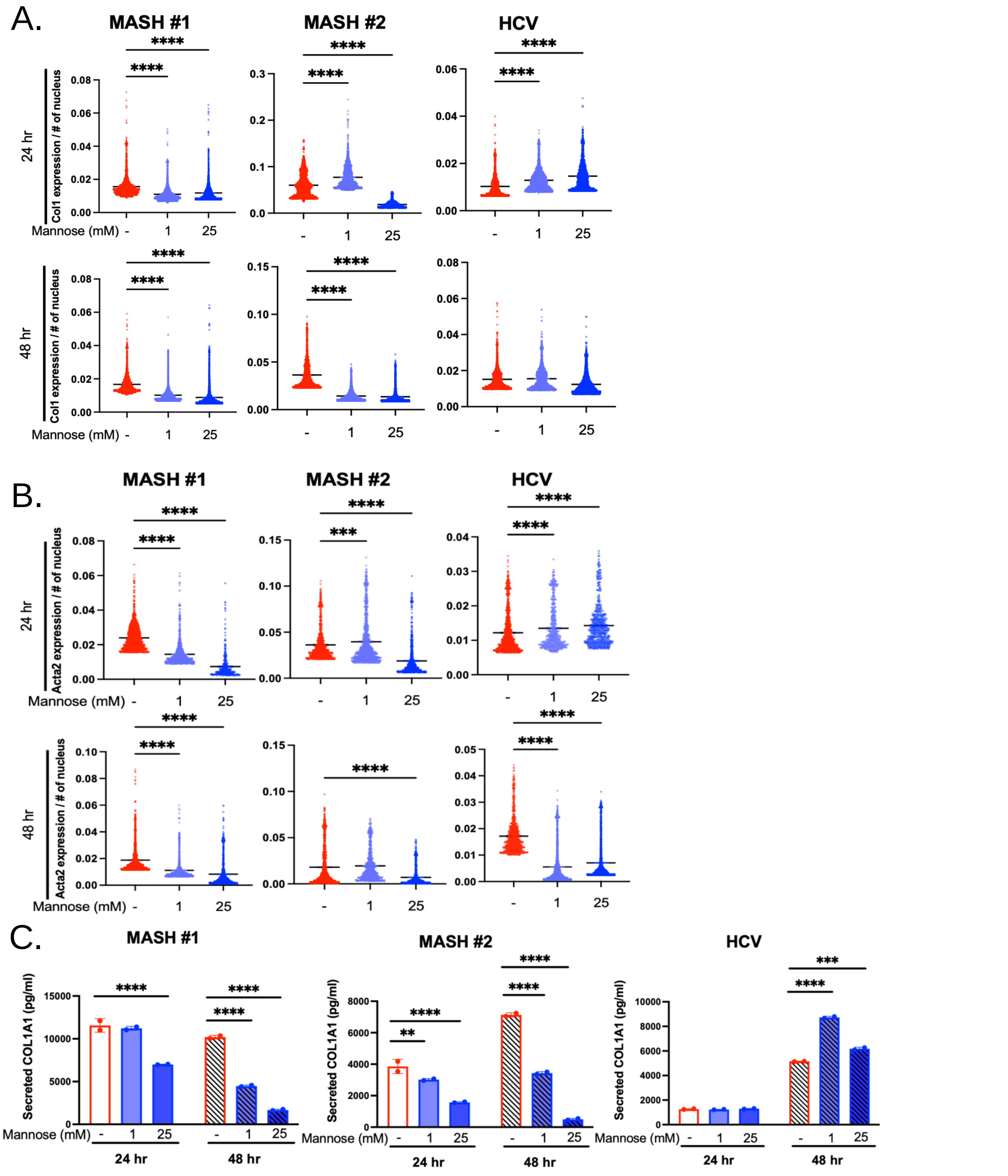

Supplemental Figure S9. Fibrogenic gene expression in human MASH PCLS with mannose treatment. Quantifications of (A) COL1 and (B) ACTA2 immunofluorescence, normalized by number of nuclei. (C) Bar graph showing secreted COL1A1 measurements. Statistical comparisons are by one sample t-test and two-way ANOVA with Dunnett's post hoc test for multiple comparisons (*p<0.05, **p<0.01, ***p<0.001, ****p<0.0001).

SDC, Supplemental Table 1.

Supplemental Table 1. Group comparisons for steatosis - Student’s t-test p-values

	ND vs MASH 6w	ND vs MASH 12w	MASH vs 5% man prevention	MASH vs 20% man prevention	MASH vs 5% man therapy	MASH vs 20% man therapy
Fat Vacuole Normalized Count- All	0.000	0.000	0.058	0.001	0.105	0.000
Steatosis Area Ratio- All	0.000	0.000	0.078	0.000	0.012	0.000
Steatosis Area Ratio- Medium	0.001	0.000	0.079	0.001	0.123	0.000
Steatosis Area Ratio- Large	0.002	0.000	0.139	0.000	0.003	0.000
Normalized count of Medium (<18 micron, count/mm2)	0.000	0.000	0.094	0.003	0.277	0.000
Normalized count of Large (>18 micron, count/mm2)	0.002	0.000	0.101	0.000	0.009	0.000

SDC, Supplemental Table 2.

Supplemental Table 2. Group comparisons for fibrosis - Student's t-test p-values

	ND vs MASH 6w	ND vs MASH 12w	MASH vs 5% man prevention	MASH vs 20% man prevention	MASH vs 5% man therapy	MASH vs 20% man therapy
Phenotypic Composite Score (Ph-FCS)	0.007	0.000	0.019	0.030	0.002	0.046
Collagen Composite Score	0.001	0.000	0.017	0.002	0.003	0.007
Morphometric Composite Score	0.015	0.000	0.017	0.023	0.003	0.032
Architecture Composite Score	0.168	0.012	0.514	0.522	0.045	0.190

SDC, Supplemental Table 3.

Supplemental Table 3. Patient characteristics for hPCLS

Patient ID	Age (years)	Sex	Disease background	Pathological diagnosis	METAVIR Fibrosis stage	Ischemia time (hours)
MASH #1	59	Male	MASH-HCC	Cirrhosis with thin acellular fibrous septa and mild steatohepatitis (Grade 0)	F4	3
MASH #2	58	Male	MASH	Dense periportal fibrosis with hyperplastic hepatocyte nodules with glycogenated nuclei	F4	4
HCV	69	Male	HCV-HCC	Chronic HCV infected cirrhosis without inflammation	F4	4

SDC, Supplemental Table 4.

Supplemental Table 4. Primer sequences for qPCR

Gene	Species	Primers
ACTA2	Human	Forward: AGATCAAGATCATTGCCCC<br>Reverse: TTCATCGTATTCCTGTTTGC
COL1A1	Human	Forward: GGCTTCCCTGGTCTTCCTGG<br>Reverse: CCAGGGGGTCCAGCCAAT
Col1a1	Mouse	Forward: GTCCCTGAAGTCAGCTGCATA<br>Reverse: TGGGACAGTCCAGTTCTTCAT
KHK	Human	Forward: GGGGCTTGTATGGTCGTGTGAG<br>Reverse: CCACCTGGCACCCGAATCTC
Khk	Mouse	Forward: ATTCTGCACGCCTACAGCTTC<br>Reverse: TACGGGAGCCATTGGAGTTG
PPIA	Human	Forward: ATGGTTCCCAGTTTTTCATC<br>Reverse: CTCCACAATATTCATGCCTTC
Ywhaz	Mouse	Forward: GAAAAGTTCTTGATCCCCAATGC<br>Reverse: TGTGACTGGTCCACAATTCCTT

SDC, Supplemental Table 5.

Supplemental Table 5. Antibody information

Antibody	Dilution	Supplier	Catalog number
**Western blot**			
**Anti-KHK**	1:1,000 (2% milk/TBST)	ThermoFisher	PA5-29004
**Anti-Collagen-1**	1:1,000 (2% milk/TBST)	Bioss	bs-10423R
**Anti-β-Actin**	1:2,000 (2% milk/TBST)	GeneTex	GTX629630
**Anti-α-Tubulin**	1:2,000 (2% milk/TBST)	DSHB	12G10
**Immunofluorescence**			
**Anti-KHK**	1:100 (1% BSA/PBS)	ThermoFisher	PA5-29004
**AlexaFluor-488**	1:2,000 (1% BSA/PBS)	ThermoFisher	A-11008
